# Development and Validation of Spectrofluorimetric Method for Determination of Biotin in Bulk and Pharmaceutical Preparations *via* its Oxidation with Cerium (IV)

**Published:** 2010-09

**Authors:** M. I. Walash, M. Rizk, Z. A. Sheribah, M. M. Salim

**Affiliations:** *Department of Analytical Chemistry, Faculty of Pharmacy, Mansoura University, Mansoura, Egypt*

**Keywords:** spectrofluorimetry, biotin, cerium (IV), oxidation, pharmaceutical preparations

## Abstract

A simple and sensitive spectrofluorimetric method was developed for the determination of biotin in pure form and in pharmaceutical preparations. The proposed method is based on the oxidation of the drug with cerium (IV) ammonium sulfate in acidic medium. The fluorescence of the produced Cerium (III) was measured at 365 nm after excitation at 255 nm. The different experimental parameters affecting the development and stability of the reaction were carefully studied and optimized. The method is applicable over the concentration range of 30-120 ng/mL with correlation coefficient of 0.9998. The detection limit (LOD) of biotin was 2.41 ng/mL while quantitation limit (LOQ) was 7.29 ng/mL. The proposed procedure was successfully applied for the determination of biotin in pharmaceutical preparations with mean recoveries of 99.55 ± 0.83 and 101.67 ± 1.53 for biotin ampoules and capsules, respectively. The results obtained were in good agreement with those obtained using the official method.

## INTRODUCTION

**Figure 1:**
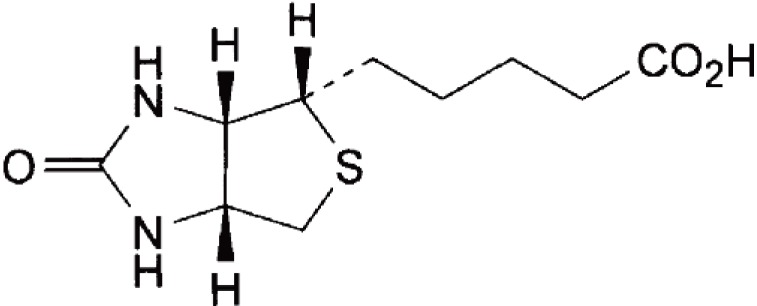
Structural formula of biotin.

Biotin, Hexahydro-2-oxo-1 H-thieno (3,4-d) imidazole -4-pentanoic acid (Figure 1), is also known as vitamin H or Co-enzyme R (1). It is a water-soluble vitamin belonging to the B-complex. It acts as a cofactor responsible for the carbon dioxide transfer in several carboxylase enzymes. It is involved in the biosynthesis of fatty acids, gluconeogenesis, and energy production, the metabolism of the branched chain amino acids and the *De Novo* synthesis of purine nucleotides. Recent research indicates that biotin plays a role in gene expression and that it may act in DNA replication (2).

The United States Pharmacopoeia (USP) (3) recommends an acid-base titration of biotin with 0.1 N NaOH using phenolphthalein as indicator. On the other hand, the British Pharmacopoeia (BP) (4) reports a non-aqueous potentiometric titration of the studied drug with 0.1 M tetrabutylammonium hydroxide.

Various methods have been published for determination of biotin either *per se* or in pharmaceutical preparations including: spectrophotometry (5, 6), spectrofluorometry (7), electro-analysis (8-10) and High Performance Liquid Chromatography (HPLC) (11-17), Liquid Chromatography-Mass Spectrometry (LC-MS) (18, 19), capillary zone electrophoresis (20, 21), Microbiological methods (22), Bioassays (23), Radioisotopic binding assays (24-26) and Non-Radioisotopic binding assays (27-32).

Since the official and reported methods for determination of the studied drug were found to be laborious, expensive and time consuming, the aim of this work was to develop a new spectrofluorimetric method for determination of biotin, that is, more sensitive, simple, rapid and less expensive than the reported and official methods. The suggested spectrofluorimetric method depends simply on oxidation of biotin using Ce(IV) in sulphuric acid medium and the relative fluorescence intensity of the formed Ce(III) was monitored at *λ*ex.=255 nm and *λ*em.=365 nm.

Cerium(IV) ion has been used as an oxidizing agent for analysis of many pharmaceutical compounds by spectrophotometric (33, 34), spectrofluorimetric (35, 36) methods or both of them (37), and chemiluminscence(38, 39).

## EXPERIMENTAL

### Apparatus

The fluorescence spectra and measurements were carried out using a Perkin-Elmer (U.S.A.) UK model LS 45 B luminescence spectrometer, equipped with a 150 Walt Xenon arc lamp, grating excitation and emission monochromators for all measurements and a Perkin-Elmer recorder. Slit widths for both monochromators were set at 10 nm. A 1 cm quartz cell was used.

### Reagents and materials

All reagents were of analytical grade and the water was always double distilled water.

- Biotin was kindly provided from Pharco Pharmaceuticals (Alex. - Egypt), its purity was 99.02 % which determined by applying the official method (3).
- Biotin Forte capsules (labeled to contain 5 mg biotin/capsule, batch # 7433017) were manufactured in Unipharma, El Obour City, Cairo, Egypt.
- Biotine Bayer 0.5% inj. (labeled to contain 5 mg biotin/ampoule, batch # F0120) was manufactured by Bayer Sante Familiale, Les Moulineaux Cedex 9, France.
- Cerium(IV) ammonium sulfate, (BDH, Pool, UK), 5 × 10^-4^ M aqueous solution was freshly prepared in 0.75 M sulfuric acid.
- Sulfuric acid (Prolabo, France), 0.75 M aqueous solutions.
- Sodium hydroxide (0.1 M) aqueous solution, (El-Nasr Pharmaceutical Chemicals (ADWIC), Egypt).
- Methanol, Spectroscopic grade (Winlab, UK).
- Acetonitrile, Spectroscopic grade (BDH, Pool, UK).
- Dimethyl sulfoxide and Dimethyl formamide, Spectroscopic grade (Merck, Darmstadt, Germany).

### Stock solutions

A stock solution of biotin was prepared by dissolving 10.0 mg of biotin in 1 mL of 0.1 M NaOH and then was completed to 100 ml with distilled water. Working standard solutions were prepared by subsequent dilution to 100 ml with distilled water. The standard solutions were found to be stable for one week when kept in the refrigerator at 4°C.

### Procedures

**Construction of calibration graphs.** Accurately measured aliquots of biotin working standard solution covering the working concentration range from 30-120 ng/mL, were transferred into a series of 10 mL volumetric flasks followed by 1.0 mL of 5 × 10^-4^ M Ce(IV) solution. The flasks were heated in a thermostatically controlled water-bath at 100°C for 25 min, cooled and diluted to the mark with distilled water. A blank experiment was performed simultaneously. The relative fluorescence intensity (RFI) of the solutions was measured at 365 nm after excitation at 255 nm. The observed fluorescence was corrected by subtracting the fluorescence intensity measured using a reagent blank. The corrected FI was plotted *versus* final concentration of the drug (ng/mL) to get the calibration graph; alternatively, the corresponding regression equation was derived.

**Procedure for the pharmaceutical preparations.** An accurately weighed quantity of the mixed contents of 10 capsules or accurately measured volume of the injection solution equivalent to10.0 mg of the drug transferred into a small conical flask and extracted or diluted with 3 × 30 ml of distilled water, respectively. One milliliter of 0.1 M NaOH was firstly added. The extract was sonicated for 15 minutes and filtered if necessary into 100 ml volumetric flask. The conical flask was washed with several mLs of distilled water. The washings were passed into the same volumetric flask and completed to the mark with the same solvent. The above procedure was then followed. The nominal content was calculated either from the previously plotted calibration graph or using the corresponding regression equation.

## RESULTS AND DISCUSSION

Cerium(IV) ion is an oxidizing agent; its acidic solution is yellow in color and has a maximum absorbance at 317 nm. Its reduced form, cerium (III), is colorless and possesses native fluorescence at λem.=365 nm (λex.=255 nm).

**Figure 2:**
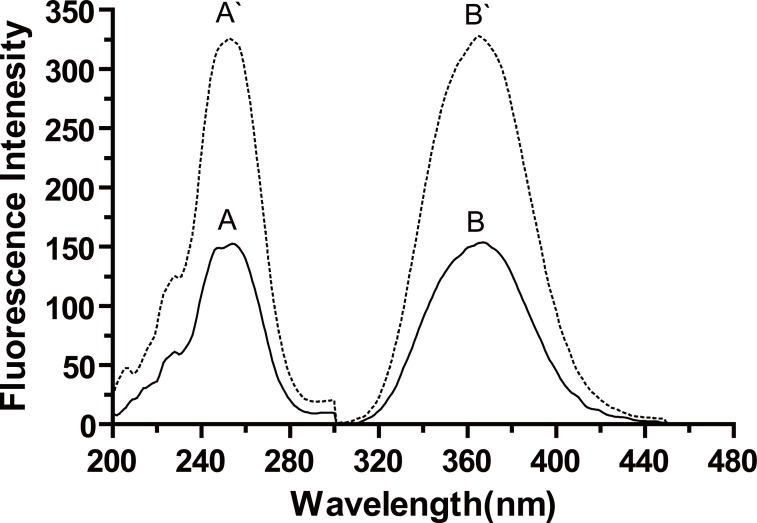
Excitation and emission spectra induced by oxidation of biotin with Ce(IV): (A, B), Blank Ce(IV) (5 × 10^-4^ M) in 0.75 M H_2_SO_4_; (A', B'), Reaction product of 50 ng/mL biotin.

Oxidation of biotin with Ce(IV) in an acid medium yields a highly fluorescent Ce(III) which exhibits maximum fluorescence at 365 nm after excitation at 255 nm (Figure 2). The oxidation product was found not to be fluorescent. This confirmed the fluorescence induced in the oxidation of the investigated drug with Ce(IV) was not attributed to its oxidation product; however, it was mainly due to the formation of Ce(III).

### Optimization of the reaction conditions

The spectrofluorometric properties of the reaction product as well as the different experimental parameters affecting the fluorophore development and its stability were carefully studied and optimized. Such factors were changed individually while the others were kept constant. These factors included the Ce(IV) concentration, type of acid and its concentration, heating time, temperature and diluting solvents.

### Effect of Ce(IV) concentration

**Figure 3:**
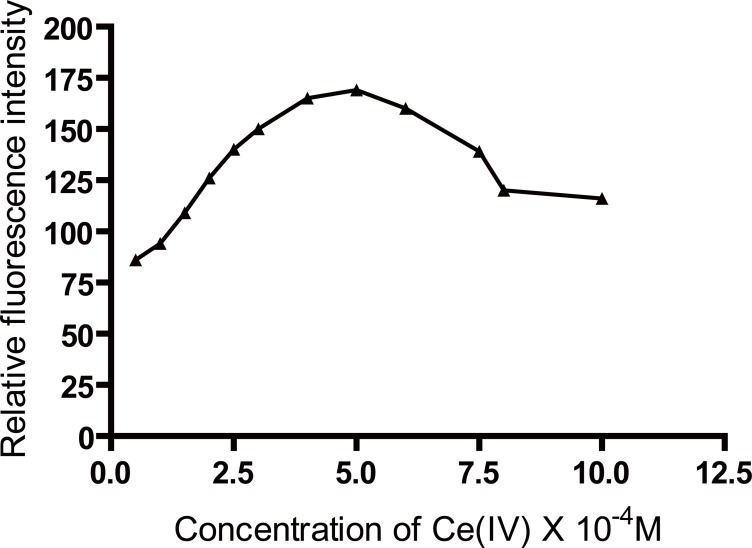
Effect of Ce(IV) concentration(M) on the fluorescence intensity induced due to oxidation of 50 ng/mL of biotin.

**Figure 4:**
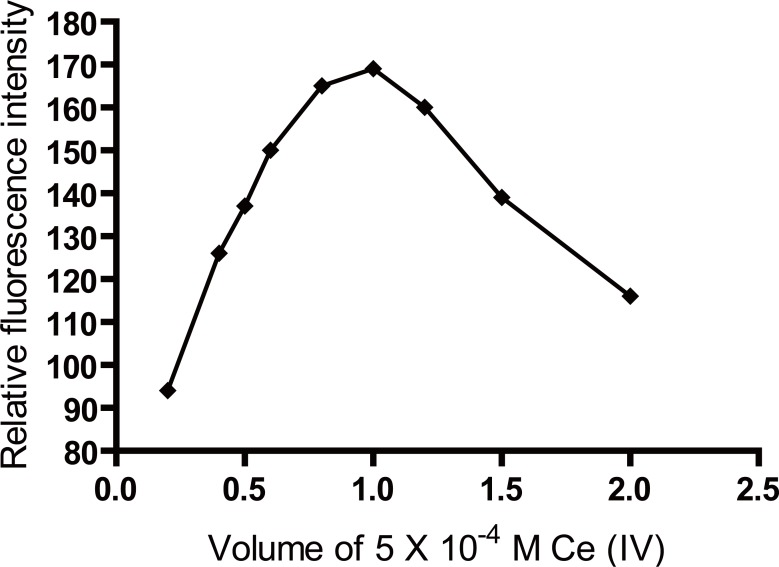
Effect of volume of 5 × 10^-4^ M Ce(IV) on the fluorescence intensity induced due to oxidation of 50 ng/mL of biotin.

The influence of Ce(IV) concentration on the fluorescence intensity of the reaction product was studied using increasing volumes of 5 × 10^-4^ M Ce(IV) solution. It was found that maximum and constant fluorescence intensity was attained using 1 mL of 5 × 10^-4^ M Ce(IV) solution (Figure 3 and Figure 4).

### Effect of acid type and its concentration

**Figure 5:**
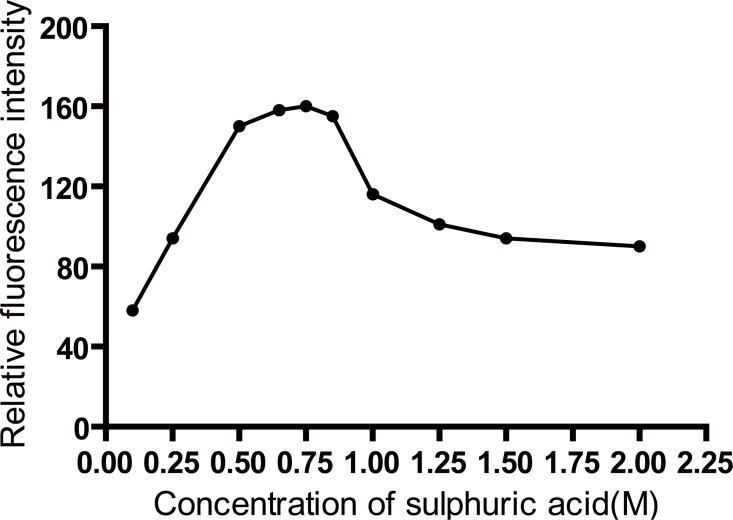
Effect of sulphuric acid concentration (M) on the fluorescence intensity induced due to oxidation of 50 ng/mL of biotin.

The oxidation reaction of Ce(IV) has to be performed in acid medium to prevent precipitation of Ce(OH)_3_. Different acids such as sulfuric acid, hydrochloric acid, nitric acid and perchloric acid were tested to determine the most suitable acid for the reaction. Nitric acid is not preferred to be used owing to the inhibitory effect of nitrate ions on the fluorescence of Ce(III).In the presence of hydrochloric acid, perchloric acid and sulfuric acid the reaction rate and the fluorescence of Ce(III) were found to be high. However, hydrochloric acid and perchloric acid gave high blank readings, so sulfuric acid was selected for the study. The effect of sulfuric acid concentration on the fluorescence intensity was studied using concentrations ranging from 0.1 to 2 M of sulfuric acid (Figure 5). It was found that the relative fluorescence intensity increased by increasing sulfuric acid concentration up to 0.75 M. So, this was used as the optimum concentration of sulfuric acid throughout the study.

### Effect of temperature and heating time

**Figure 6:**
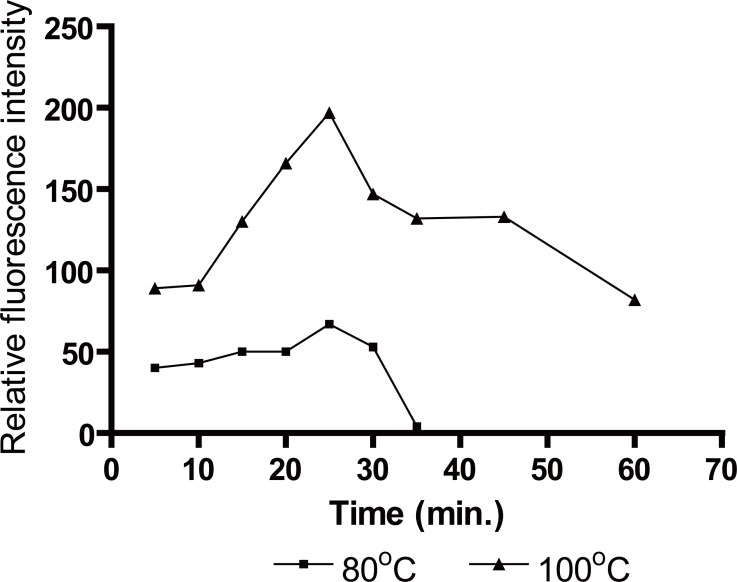
Effect of heating time on fluorescence intensity induced by Ce(IV) after oxidation of 50 ng/mL of biotin.

Oxidation of the studied drug with Ce(IV) was carried out at different temperature sets ranging from 25-100°C for various periods of time ranging from 5 to 60 min (Figure 6). At 25°C, the reaction proceeds slowly. However, heating the reaction solution was found to increase both reaction rate and the fluorescence intensity. The results revealed that the optimum temperature was 100°C. Complete reaction was attained upon boiling for 25 min, and a longer heating time decreased the relative fluorescence intensity.

### Effect of diluting solvents

Dilution with different solvents such as water, methanol, acetonitrile, dimethyl sulfoxide and dimethyl formamide was attempted. It was found that water was the best solvent for dilution as it gave the highest fluorescence intensities and the lowest blank reading. A distinct and sharp decrease in the fluorescence intensities was attained upon using acetonitrile and methanol, while dimethyl sulfoxide and dimethyl formamide quench the fluorescence completely.

## VALIDATION OF THE METHOD

### Accuracy and precision

The proposed method was evaluated by calculating the accuracy as percent relative error (% Error) and precision as percent relative standard deviation (RSD %). The data obtained are abridged in Table 1.

**Table 1:** Performance data of the proposed method

Parameter	Biotin

Concentration range (ng/mL)	30-120
Limit of detection (LOD) (ng/mL)	2.41
Limit of quantification (LOQ) (ng/mL)	7.29
Correlation coefficient (r)	0.9998
Slope	2.68
Intercept	-13.05
Standard deviation of the residuals, S_y/x_	1.94
Standard deviation of the intercept, S_a_	1.95
Standard deviation of the slope, S_b_	0.02
Relative standard deviation, % RSD	1.20
Percentage error, % Error	0.49

Statistical analysis (42) of the results, obtained by the proposed and the official method (3) using Student’s t-test and variance ratio F-test, shows no significant difference between the performance of the two methods regarding the accuracy and precision, respectively (Table 2). The official method involved acid-base titration of biotin using 0.1 N NaOH as a titrant and phenolphthalein as indicator (3).

**Table 2:** Application of the proposed and official methods to the determination of biotin in pure form

Compound	Proposed method			Official method (3)
	Concentration taken (ng/mL)	Concentration found (ng/mL)	Recovery (%)	Recovery (%)

Biotin	30.0	29.46	98.21	98.04
	40.0	40.02	100.05	100.98
	60.0	61.03	101.72	98.04
	80.0	80.02	100.02	
	100.0	99.03	99.03	
	120.0	120.40	100.34	
Mean ± S.D.			99.89 ± 1.197	99.02 ± 1.69
Student’s t-test			0.91 (2.36)	
F-test			2.01 (19.35)	

Each result is the average of three separate determinations. Values in parentheses are the tabulated t and F values, respectively at *p*=0.05 (42).

**Repeatability.** The repeatability and reproducibility (intra-day precision) was evaluated through replicate analysis of biotin authentic sample spiked with 60.0 ng/mL on three successive times on the same day. The mean percentage recoveries based on the average of three separate determinations were 100.38 ± 1.61 as shown in Table 3.

**Table 3:** Validation data of the proposed method for the determination of biotin in pure form

Regimen	Parameters	Recovery %		
		Amount Added (ng/mL)	Amount Found (ng/mL)	Recovery %

Intra-day		60.0	61.21	102.02
		60.0	59.28	98.80
		60.0	60.20	100.34
	(x)			100.38
	± S.D.			1.61
	% RSD			1.60
	% Error			0.65
Inter-day	1^st^ day	100.0	99.03	99.03
	2^nd^ day	100.0	102.09	102.09
	3^rd^ day	100.0	101.98	101.98
	(x)			101.03
	± S.D.			1.74
	% RSD			1.72
	% Error			0.70

**Intermediate precision.** The Intermediate precision (inter-day precision) was evaluated through replicate analysis of Biotin spiked with 100.0 ng/mL on three successive days. The percentage recoveries based on the average of three separate determinations were 101.03 ± 1.74 (Table 3).

### Concentration ranges and calibration graphs

After optimizing the reaction conditions, a linear calibration graph was obtained over the range of 30-120 ng/mL. Analysis of the data gave the following regression equation:

F=−13.05+2.68 C  r=0.9998

where F is fluorescence intensity, C is the concentration of the drug in ng/mL and r is correlation coefficient.

**Figure 7:**
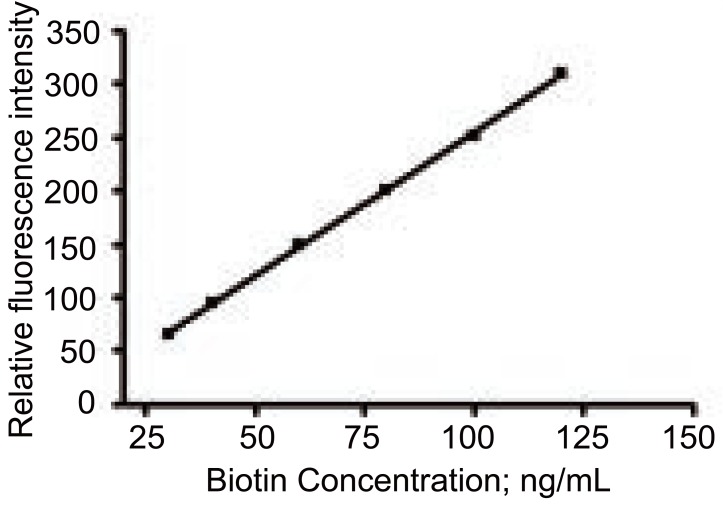
Calibration graph of biotin determined by the proposed method.

The calibration graph adopting the proposed method is shown in Figure 7.

Validation of the method was evaluated by statistical analysis (42) of the regression data regarding standard deviation of the residuals (S_y/x_), the standard deviation of intercept (S_a_), and standard deviation of the slope (S_b_). The data obtained are included in Table 1. The small values given point out to the low scattering of the points of the calibration curve.

### Limit of quantitation and limit of detection

The limit of quantitation (LOQ) was determined by establishing the lowest concentration that can be measured according to ICH Q2) R1) recommendation (41) below which the calibration graph is non linear and was found to be 7.29 ng/mL.

The limit of detection (LOD) was determined by evaluating the lowest concentration of the analyte that can be readily detected and was found to be 2.41 ng/mL.

LOQ and LOD were calculated according to the following equations (41):

LOQ = 10 S_a_/b

LOD = 3.3 S_a_/b

where S_a_ is the standard deviation of the intercept of regression line, and b is the slope of the calibration curve.

### Robustness of the method

The robustness of the proposed method is demonstrated by the constancy of the fluorescence intensity with minor changes in the experimental parameters such as 5 × 10^-4^ M Ce(IV) volume, 1.0 ± 0.1 mL and change in the concentration of sulfuric acid, 0.75 ± 0.1 M. These minor changes that may take place during the experimental operation did not greatly affect the fluorescence intensity.

### Pharmaceutical Applications

The proposed method was applied to the determination of biotin in its dosage forms. The selectivity of the method was investigated by observing any interference encountered from the capsule excepients. These excepients did not interfere with the proposed method (Table 4). The results of the proposed method were statistically compared with those obtained using the official method. The latter method involved HPLC determination of biotin in its dosage forms. The liquid chromatograph is equipped with a 200-nm detector and a 4.6 mm × 15 cm column containing 3 μm packing L7. The flow rate is about 1.2 mL per minute (3).

Statistical analysis (42) of the results obtained using Student’s t-test and variance ratio F-test revealed no significant difference between the performance of the two methods regarding the accuracy and precision, respectively (Table 4).

**Table 4:** Application of the proposed method to the determination of biotin in its pharmaceutical preparations

Compound	Proposed method			Official method (3)
	Concentration taken (ng/mL)	Concentration found (ng/mL)	Recovery (%)	Recovery (%)

Biotine Bayer 0.5% inj^a^	60.0	60.30	100.50	102.09
(Biotin 5 mg/mL)	80.0	79.23	99.04	99.98
Batch # F0120	100.0	99.10	99.10	100.70
Mean ± S.D			99.55 ± 0.83	100.92 ± 1.07
% RSD			0.83	1.062
%Error			0.48	0.613
Student’s t-test			1.64 (2.78)	
F-test			2.50 (19.00)	
Biotin forte capsules^b^	60.0	60.00	100.00	98.96
(Biotin 5 mg/capsule)	80.0	81.60	102.00	99.93
Batch # 7433017	100.0	103.00	103.00	99.45
Mean ± S.D			101.67 ± 1.53	99.44 ± 0.48
% RSD			1.50	0.487
% Error			0.87	0.281
Student’s t-test			0.42 (2.78)	
F-test			12.25 (19.00)	

Each result is the average of three separate determinations. Values in parentheses are the tabulated t and F values, respectively at *p*=0.05 (42).

a Product of Bayer Sante Familiale, Les Moulineaux Cedex 9, France;

b Product of Unipharma, El Obour City, Cairo, Egypt.

### Stoichiometry of the reaction

**Figure 8:**
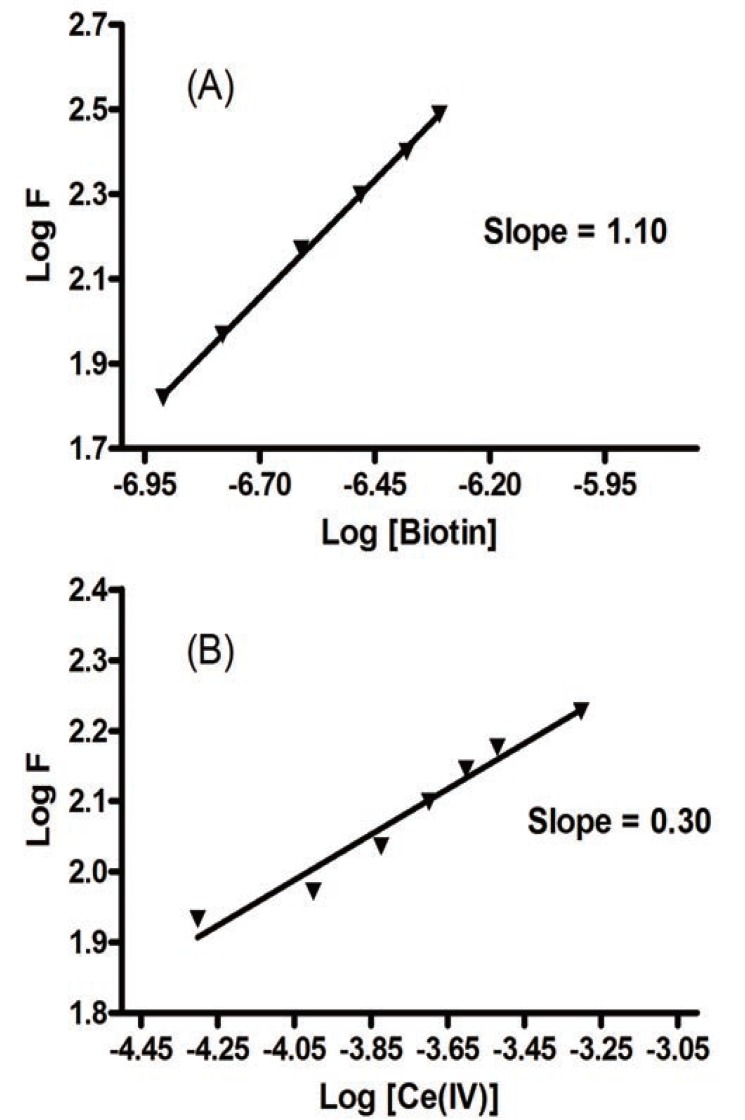
Stoichiometry of the reaction between biotin and Ce(IV) adopting limiting logarithmic method. (A), Log [biotin] *vs* log F; (B), Log [Ce(IV)] *vs* log F.

Figure 8 shows the stoichiometry of the reaction between the studied drug and cerium(IV) adopting the limiting logarithmic method (40). The fluorescence intensity of the the Ce(III) was alternatively measured in the presence of excess Ce(IV) and the studied drug. Plots of log [drug] vs log F and log [Ce(IV)] vs log F gave straight lines, the values of the slopes were 0.30:1.10 (Ce(IV): drug). Hence, it is concluded that the molar reactivity of the reaction is 4:1, i.e. the reaction proceeds in a ratio of 4:1.

### Mechanism of the reaction

**Figure 9:**
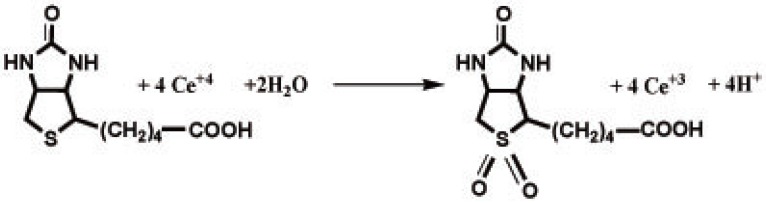
The proposal for the reaction pathway between biotin and Ce (IV).

Based on the observed molar reactivity of the reaction of the drug with Ce^+4^ and by analogy to previous reports (5), it is assumed that the -S- group in biotin is oxidized to the corresponding sulphone. Proposal for the reaction pathway are shown in Figure 9.

## CONCLUSION

The present work describes a validated spectrofluorometric method for the determination of the studied drug without interference from common excipients. Hence, it may be applied for the routine quality control of the studied drug either in bulk or in its corresponding dosage forms. The methodology appears to be straight forward and results are relevant. Another advantage is that compared to the existing reported methods for determination of this drug, it is several times more sensitive. From an economic point of view, the proposed method is simple, rapid and inexpensive. The use of water as diluting solvent is a further advantage. In sum, we have presented an economic alternative method to other official and reported methods and to high cost HPLC methods.
